# A Systematic Review and Meta-Analysis of Creep Feeding Effects on Piglet Pre- and Post-Weaning Performance

**DOI:** 10.3390/ani13132156

**Published:** 2023-06-30

**Authors:** Bruno B. D. Muro, Rafaella F. Carnevale, Matheus S. Monteiro, Renjie Yao, Felipe N. A. Ferreira, Clarice S. S. Neta, Francisco A. Pereira, Dominiek Maes, Geert P. J. Janssens, Glen W. Almond, Cesar A. P. Garbossa, Tatiane T. N. Watanabe, Diego F. Leal

**Affiliations:** 1 Department of Nutrition and Animal Production, School of Veterinary Medicine and Animal Science, University of São Paulo (USP), Pirassununga 13635-900, SP, Brazil; bruno.muro@usp.br (B.B.D.M.); rafaella.carnevale@usp.br (R.F.C.); francisco.pereira@agroceres.com (F.A.P.); cgarbossa@usp.br (C.A.P.G.); 2 Nerthus Pesquisa e Desenvolvimento LTDA, São Carlos 13563-651, SP, Brazil; matheus.salibamonteiro@gmail.com; 3 Department of Internal Medicine, Reproduction and Population Medicine, Faculty of Veterinary Medicine, Ghent University, 9000 Ghent, Belgium; renjie.yao@ugent.be (R.Y.); dominiek.maes@ugent.be (D.M.); 4 Department of Veterinary and Biosciences, Faculty of Veterinary Medicine, Ghent University, 9000 Ghent, Belgium; geert.janssens@ugent.be; 5 Agreceres Multimix Nutrição Animal LTDA, Rio Claro 13502-741, SP, Brazil; felipe.alves@agroceres.com (F.N.A.F.); clarice.silva@agroceres.com (C.S.S.N.); 6 Department of Population Health and Pathobiology, College of Veterinary Medicine, North Carolina State University (NCSU), Raleigh, NC 27606, USA; gwalmond@ncsu.edu (G.W.A.); tnegrao@ncsu.edu (T.T.N.W.)

**Keywords:** creep feeding, piglet, growth performance, lactation, weaning

## Abstract

**Simple Summary:**

In pig production, nursing piglets are frequently offered a highly palatable and easily digestible diet (creep feed). Creep feeding is believed to increase the weaning weight of piglets and to facilitate a smooth transition from sow’s milk to the dry feed. However, the research results are conflicting, and this might be due to the fact that several factors can impact the positive aspects of creep feeding during lactation. Therefore, the aim of the present study was to evaluate the effects of creep feeding on piglet pre- and post-weaning performance. Our results demonstrated that providing piglets with creep feed in lactation increases piglet body weight at weaning and post-weaning growth performance. We also identified that a minimum of 14 days of creep feed provision is necessary to realize a higher body weight at weaning.

**Abstract:**

In the present systematic review and meta-analysis, we evaluated the effects of providing piglets with creep feed during lactation on piglet pre- and post-weaning performance. A total of 20 articles met the inclusion criteria. Creep feeding in lactation improved pre-weaning piglet performance in 46% of the studies selected, while 58% of the included studies reported that creep feeding in lactation improved piglet performance during the nursery phase. Creep feeding increased the average piglet body weight (creep = 7.23 ± 0.30, no creep = 6.96 ± 0.31; *p* = 0.03) and litter weight (creep = 81.2 ± 4.18, no creep = 76.4 ± 4.22; *p* < 0.001) at weaning. The average piglet body weight and litter weight were positively associated (*p* < 0.001 and *p* < 0.001, respectively) with total creep feed intake. Creep feeding of piglets for more than 14 days increased (*p* = 0.003) the litter weight at weaning compared to litters not provided or provided for shorter periods with creep feed. The present work strengthened the notion that creep feeding during lactation presents opportunities for improving weaning weights and post-weaning piglet performance compared to litters not provided or provided for shorter periods with creep feed.

## 1. Introduction

Weaning is one of the most stressful events in the life of the pig [1]. During weaning, piglets are exposed to a host of stressors, including removal from the sow, interaction with unacquainted piglets, and the transition from sow milk to a less digestible diet [2]. Under natural conditions, the process of weaning takes several weeks [3]. During this period, piglets have the opportunity to be familiarized with solid food while suckling frequencies decrease gradually [4]. In modern pig husbandry, however, piglets are weaned at an early age, typically three to five weeks of age, when nutrients and energy are still obtained from the sow’s milk [1]. After weaning, piglets have to rely on a less-digestible plant-based diet when they still have reduced digestive enzymatic activity for these ingredients [5]. This often results in intestinal dysfunctions, leading to decreased health, feed intake, and growth performance [6].

Creep feed is a highly palatable and easily digestible diet that is frequently offered to piglets while they are still nursing [7]. The creep feeding of piglets presents opportunities for increasing weaning weight and easing the transition from the sow’s milk to solid food, promoting a suitable transition at weaning [4,7,8]. One of the main limiting factors associated with the practice of creep feeding is that only a certain proportion of piglets (45% to 65%) within the litter consume creep feed [9]. However, there is evidence that piglets that are proven creep feed consumers have better initial post-weaning feed intake and growth performance than non-consumers [10]. Creep feed consumption can be affected by several factors, including time of onset [11], duration of creep feeding [12], type of creep feeder [4,13], physical form of the creep diet [14,15], sow’s milk production and lactation length [16], inclusion of feed additives in the creep diet [6,17], litter size [8,18], and feed complexity [19].

Research efforts have been directed toward increasing the proportion of piglets that consume creep feed, hence, improving piglet pre- and post-weaning performance. However, studies have yielded equivocal outcomes. Creep feeding during lactation had no effect on piglet pre-weaning performance in some studies [7,12,20]. In other studies, the piglets offered creep feed in lactation had an increased body weight at weaning [15,21,22]. In the same context, creep feeding was shown to increase feed intake and body weight gain early after weaning [6,14,23]. However, in other studies, creep feeding in lactation had no direct beneficial impact on post-weaning performance [24,25,26,27]. Furthermore, Lee and Kim [11] observed that the piglets started on creep feeding from the first week after birth had a higher body weight at weaning compared to piglets offered creep feed from the second and third weeks after birth. Therefore, a systematic and quantitative assessment of published studies for a comprehensive conclusion of the creep feeding effects on piglet pre- and post-weaning performance is warranted.

Based on the foregoing observations, the objective of the present study was to evaluate the effects of creep feeding on piglet pre- and post-weaning performance by conducting a systematic review and a meta-analysis of published randomized controlled studies.

## 2. Materials and Methods

The present systematic review and meta-analysis were conducted in accordance with the Preferred Reporting Items for Systematic Reviews and Meta-Analyses (PRISMA) checklist [28].

### 2.1. Research Strategy

An exhaustive database search was performed independently by two co-authors (Muro BBD, Carnevale RF) using PubMed, Web of Science, Science Direct, Scopus, and SciELO databases up to December 2022. The search terms were the following: (i) creep feeding, piglets, growth; (ii) creep feeding, sow, weight loss; (iii) creep feeding, weaning, swine; (iv) creep feeding, piglet, sow; (v) creep feeding, piglet, performance; (vi) creep feeding, sow, performance; (vii) creep feeding, piglet, sow. Only original research articles and randomized controlled trials conducted with pigs were selected, excluding books, book chapters, conference abstracts, and reviews. Only research articles written in English were chosen. Only articles published from 2000 onwards were considered eligible.

### 2.2. Selection of Studies

#### 2.2.1. Inclusion and Exclusion Criteria

The inclusion criteria were the following: (i) studies that evaluated the effects of creep feeding on piglets’ pre-weaning performance; (ii) studies that had an experimental group without creep feed; (iii) studies that were published in the English language; (iv) studies published in a peer-reviewed journal; (v) studies published from 2000 onwards (the date restriction was imposed to reduce differences regarding genetic improvements and nutrition advances). The following were the exclusion criteria: studies that only evaluated the effects of creep feeding on piglet post-weaning performance.

#### 2.2.2. Database Formation

The data were extracted from the papers by two investigators, independently. The information related to the proposed theoretical model and other additional variables were copied from the selected publications and transferred to an electronic spreadsheet. The methodology used for database formation and coding was performed according to Lovatto et al. [29] and Sauvant et al. [30]. The following data were extracted from each individual reference: the year of publication, the country in which each study was conducted, the journal name, the total number of piglets evaluated in the study, the average number of piglets per litter, the weaning age, the time of onset of creep feeding, the duration of creep feeding, the piglet body weight at weaning, the litter weight at weaning, the the total feed intake of the litter, individual piglet feed intake (average), the piglet daily gain (average), and piglet mortality.

#### 2.2.3. Quality Criteria

A quality appraisal of the articles selected was carried out as previously described by Muro et al. [31]. Scores of 1 (partially adequate) or 2 (adequate) were assigned to specific scientific criteria. The maximum attainable value was 24 points. The following were the quality parameters:

i. Randomization: randomized studies scored 2, while a non-randomized study, or one where this was not clearly stated, scored 1;
ii. Breed or genetic line: studies that mentioned breed or genetic line received a score of 2, and when it was not clearly stated, they scored 1;
iii. Environmental characterization: studies that provided information regarding temperature and humidity in the farrowing room scored 2, and those that did not describe it scored 1;
iv. Litter size: studies that described the mean litter size in each experimental group scored 2, and those that did not mention it scored 1;
v. Composition of the creep diet: studies that detailed the ingredients and the nutritional composition of the creep diet scored 2, and those that did not provide this information scored 1;
vi. Creep feeder placement: studies that described where the feeder was located in the farrowing crate scored 2, and those that did not provide this information scored 1;
vii. Type of creep feeder: studies that described the type of creep feeder scored 2, and those that did not describe it scored 1;
viii. Sample size: studies using more than 30 sows per treatment scored 2 and those using less than 30 sows per treatment scored 1;
ix. Parity: studies that specified parity scored 2, while those that did not clearly mention it scored 1;
x. Piglet body weight at the start of the experiment: studies that stated the piglet body weight at the start of the experiment scored 2, and those that did not scored 1;
xi. Post weaning performance: studies that evaluated piglet post-weaning performance scored 2, and those that did not scored 1;
xii. Physical form of the creep feed: trials that described the physical form of the creep feed scored 2; and trials that did not describe it scored 1;

### 2.3. Statistical Analysis

The statistical analyses were performed in R software (R Core Team, version 4.2.0, Vienna, Austria). The meta-analysis was performed following three sequential analyses as follows: graphical (to control database quality and to observe biological coherence of data), principal component analysis (PCA—to identify related factors among all variables), and multilevel statistical models (for variance analysis).

The PCA was applied to the data with scaling in order to visually analyze the relation among variables associated with: (i) creep feeding (i.e., duration of creep feeding, age of piglets at the start of creep feeding, total creep feed intake, and average daily feed intake); (ii) piglet/litter performance during lactation (i.e., body weight at weaning, average daily gain, and litter weight at weaning); and (iii) piglet/litter traits (i.e., weaning age and litter size). Only creep-fed piglets were included in this analysis. The effect of creep feeding in lactation on piglet pre- and post-weaning performance was evaluated in the systematic review. In the meta-analysis, only the effects of creep feeding on pre-weaning performance were evaluated.

For the multilevel statistical analysis, the assumption of normality and homogeneity of variances were graphically evaluated (i.e., histogram, normal probability plot of residuals) and tested by Shapiro–Wilk and Barlett, respectively. The data were presented as mean ± SEM, and the results were considered significant at *p* < 0.05.

The effects of creep feeding on litter weight and average piglet body weight at weaning were analyzed by linear multilevel models fitted by a normal distribution. The coding of publications and weaning age were considered as random effects. Linear multilevel models were also used to investigate the association between the average piglet’s body weight and litter weight at weaning and total creep feeding disappearance. The coding of publications and weaning age were considered as random effects. In order to observe the impact of the duration of creep feeding on piglets’ performance, groups were created according to graphical observation. The litter weight and average piglet body weight at weaning were considered to be indicative of successful creep feeding, and treatment duration was a factor potentially affecting the total creep feed intake during lactation. With the objective to determine the categorical groups, a non-linear model using generalized least square (GLS) was used, where litter weight was used as a dependent variable and the duration of creep feeding was used as an independent variable. The GLS analysis indicated that the creep feeding effect plateaued at day 14 of provision. The predicted model indicated that the total creep feed disappearance increased linearly (*p* < 0.001) with the duration of creep feeding after 14.04 days of creep feeding (Supplementary Materials Figure S1). Therefore, to observe the impact of creep feeding on litter performance, three groups were considered: no creep (i.e., piglets not provided with creep feed), Creep 1–13 (i.e., piglets provided with creep feed for 1–13 days); Creep 14+ (i.e., piglets provided with creep feed for at least 14 days).

## 3. Results

### 3.1. Systematic Review

**Figure 1:**
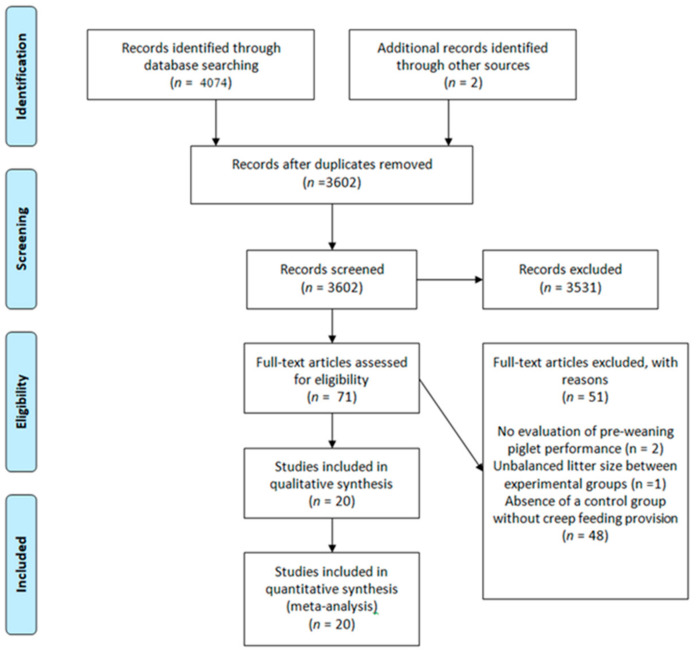
Preferred reporting items for systematic reviews and meta-analyses (PRISMA) flow diagram of studies identified and included for qualitative and quantitative synthesis of studies investigating the effects of creep feeding on piglet performance.

The selection steps of studies are shown in the PRISMA flowchart (Figure 1). The initial search yielded 4074 unique studies. After the exclusion of duplicates, title and abstract evaluation, and articles not fulfilling the inclusion criteria, 71 articles were considered eligible for full-text evaluation. Two eligible articles [32,33] were identified through reference list screening. The absence of an experimental group without the provision of creep feed was the reason for the exclusion of 48 articles. Two articles were excluded because there was no evaluation of creep feeding on piglet pre-weaning performance. One study was excluded due to the unbalanced litter size among experimental groups. Thus, a total of 20 articles were selected to compose the present work.

The quality of the selected articles according to the established criteria (min 12 and max 24) is presented in Table S1. The highest score attained was 23 [22] of a possible 24, while the minimum score attained was 16 [33]. In 60% (12/20) of the studies, the litters were assigned at random to experimental groups. Information on the breed or genetic line was stated by 85% (17/20) of the selected studies. In only one study (5%), there was available information on the ambient temperature and humidity in the farrowing room. The litter size was stated in 95% (19/20) of the studies included. In 65% (13/20) of the studies, there was detailed information on the composition of the creep feed. Creep feeder position and type were described in 25% (5/20) and 30% (6/20) of the studies, respectively. Parity was stated in 35% (7/20) of the studies included. The piglet body weight at the start of the experiment and post-weaning performance were described in 100% and 50% of studies, respectively. The physical form of the creep feed (e.g., mash, pellet, or liquid) was stated in 65% (13/20) of the studies included. Two studies evaluated the effects of duration and time of onset of creep feeding. The duration of creep feeding varied from 3–28 days, whilst the time of onset of creep feeding varied from 2–21 days post-farrowing. The number of treated (creep-fed) and control litters (non-creep-fed) included in the studies varied from 3–80 and 4–80, respectively. The average litter size varied from 9.31–13.6 for each individual reference.

The effects of creep feeding on piglet performance are presented in Table 1 and a summary of the main features of the studies included are shown in Table 2. From the studies that evaluated the effect of creep feeding of pre-weaning performance, 46% reported a positive effect whilst in 54% no effect was found. In 58% of the studies included creep feeding improved piglet performance at the nursery phase.

### 3.2. Meta-Analysis

**Figure 2:**
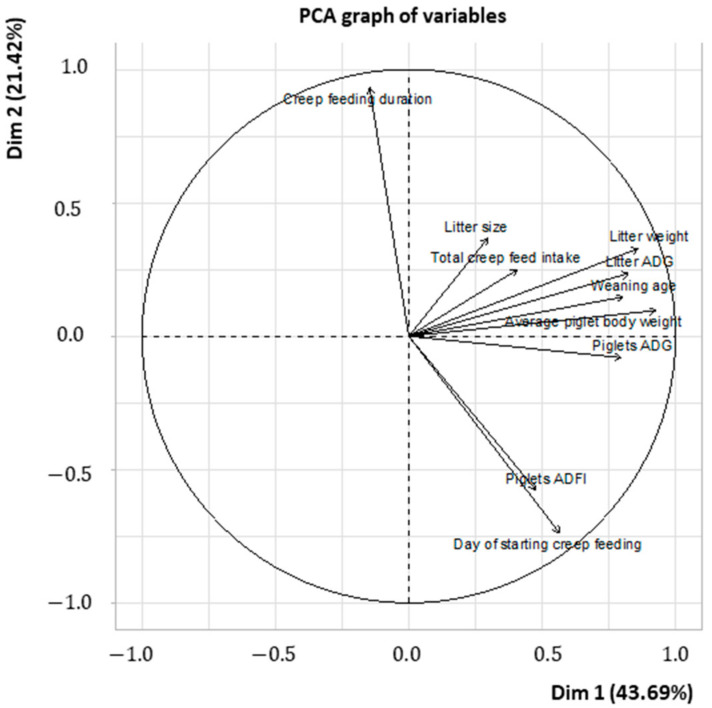
Principal component analysis among variables associated with creep feed provision and consumption, variables associated with piglet/litter performance during lactation, and variables associated with piglet/litter traits.

The descriptive data of the main variables evaluated in the meta-analysis are shown in Table 3. The first two components of the PCA jointly represent 65.11 of the total variances of the data. The first component explained 43.69% of the variance, while the second component explained 21.42%. The variables that showed the highest contribution to the first component were mainly related to piglets and litter performance (i.e., litter ADG, piglets ADG, litter weight, average piglet body weight, and weaning age). These variables were all located at the same side in the PCA plot (Figure 2). The variables related to creep feeding (i.e., duration of creep feeding and time of onset) and piglet consumption of creep feed (i.e., piglet ADFI) were the main contributors to the second component. The time of onset of creep feeding and piglet ADFI were located at the same side in the PCA plot, and they were opposite to the duration of creep feeding.

Creep feeding increased the average piglet body weight (creep = 7.23 ± 0.30, no creep = 6.96 ± 0.31; *p* = 0.03) and litter weight (creep = 81.2 ± 4.18, no creep = 76.4 ± 4.22; *p* < 0.001) at weaning (Table 4).

Both the average piglet body weight and litter weight at weaning were positively associated (*p* < 0.001 and *p* < 0.001, respectively) with total creep feed intake of the litter, as shown in Table 5.

**Figure 3:**
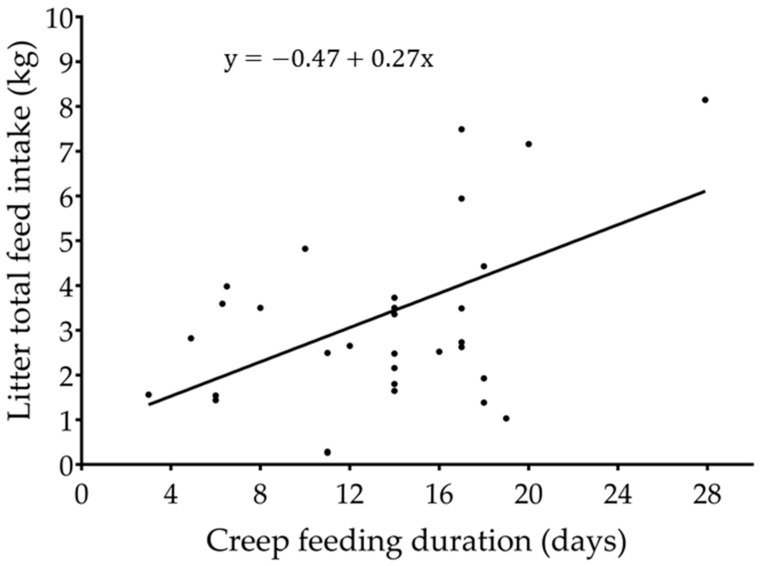
Association between litter creep feed intake and duration of creep feeding (*p* < 0.001; R^2^ = 0.40). The codification of the studies was included as a random variable.

**Figure 4:**
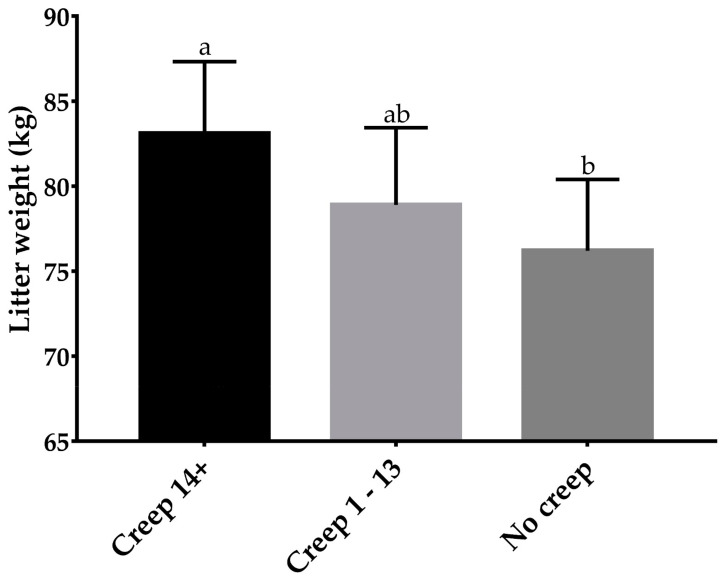
The effect of different durations of creep feeding on the litter weight at weaning; no creep: litters that did not have access to creep feed; Creep 1–14: litters that had free access to creep feed from one to 14 days; Creep 14+: litters that had free access to creep feed for 14 days or more. ^a,b^ *p* = 0.003.

The total creep feed consumption increased (*p* < 0.001; R^2^ = 0.40; Figure 3) as the creep feeding duration increased. There was no association (*p* = 0.19) between weaning age and total creep feed consumption. The litter size had no influence (*p* = 0.80) on total creep feed consumption. The creep feeding of piglets for more than 14 days increased (*p* = 0.003) the litter weight at weaning compared to litters not provided with creep feed (Figure 4).

## 4. Discussion

The present systematic review summarized and critically appraised the existing literature regarding the effects of creep feeding on piglet pre- and post-weaning performance. Although the results of creep feeding are highly variable among studies, we observed that creep feeding in lactation improved pre-weaning piglet performance in 46% of the studies selected. Likewise, 58% of the included studies reported that creep feeding in lactation improved piglet performance at the nursery phase.

Several factors can influence creep feed consumption and, hence, its beneficial effects on piglet performance. Therefore, it is important to identify the factors that could influence the results of studies looking at the impact of creep feeding. We observed that there was insufficient description in the studies regarding the type of creep feeder used. The method of creep feed presentation can influence creep feed consumption. Wattanakul et al. [4] reported that the increase in feeding space and accessibility (i.e., use of a tray) resulted in an increased feeder visiting time for the piglets and creep feed intake for the whole litter. Likewise, interactive feeders (e.g., playfeeder) stimulate the exploratory behavior of piglets, increasing feed intake [13,42]. Furthermore, there is evidence that feeder placement can influence creep feed intake. Oliveira et al. [21] investigated the influence of the creep feeder location (i.e., feeder placed at the back or at the front of the farrowing crate) on creep feed consumption; the authors noted that placing the creep feeder at the front of the farrowing crate (near the sows’ head) increased the creep feed intake, resulting in a greater growth rate.

Our systematic review identified a lack of information with respect to the ingredient composition of the creep feed, with only 35% of the studies detailing this information. This variable was shown to influence creep feed consumption, with positive impacts on piglet pre- and post-weaning performance [12,44,45,46,47]. Piglets offered a high-complexity creep diet in pellet form (30% pulverized oat groats and 25% spray-dried whey, 10% extruded soy protein concentrate, 6% spray-dried porcine plasma, 6% menhaden fish meal) had improved pre-weaning average daily gain and feed intake, and the proportion of creep feed eaters increased compared to piglets fed a simple diet in meal form (60% milo, 32% soybean meal, and 3% choice white grease). These piglets had improved post-weaning feed intake, daily gains, and weight uniformity and reduced post-weaning lag [48]. Pajor et al. [45] reported that piglets with access to the high-complexity diet consumed more creep feed before weaning than piglets offered a simple creep diet. Likewise, piglets fed a soluble creep diet formulation (20% oatmeal and 10% soy protein isolate) in gruel form were heavier at weaning, with this effect maintained through the first week of the nursery period [15]. Therefore, in order to facilitate the critical appraisal of the research results, studies assessing the effects of creep feeding on piglet performance should provide detailed information about the creep feed, as the inclusion of highly digestible and palatable ingredients in the creep diet can increase the proportion of creep feed eaters, with reflections on growth performance.

The microclimate of the farrowing room is another important factor that may influence the results of creep feeding. Nonetheless, we observed that in 95% of the studies included, there was no information regarding ambient temperature and humidity in the farrowing room. Exposure to high ambient temperature negatively affects milk production of sows [49], which, in turn, may influence creep feed consumption. Renaudeuau and Noblet [50] found a daily creep feed consumption 67% greater in litters from heat-stressed sows compared to litters at thermoneutrality (388 and 232 g/day, respectively). Future investigations looking at the impact of creep feeding should consider the ambient temperature and humidity in the farrowing room, as well as the effect of the season, as potential interference factors, avoiding, therefore, study bias.

Our meta-analysis demonstrated that the creep feeding of piglets improved both the average piglet body weight and litter weight at weaning. Furthermore, a positive association exists between piglet performance at weaning and the total creep feed disappearance. This result further supports the notion that the extent of creep feed consumption is essential to achieve an increased pre-weaning performance.

Another important result of the present meta-analysis is that the duration of creep feeding is positively associated with creep feed consumption. Our analysis demonstrated that piglets should be creep fed for a minimum of 14 days in order to achieve increased pre-weaning performance. Therefore, piglets should be started on creep feeding at the beginning of the second week of lactation when they are weaned at ≤24 days of age or in the third week of lactation when piglets are weaned at ≥28 days of age.

The results from our meta-analysis demonstrate that litter size had no influence in total creep feed disappearance. This outcome is contrary to that of Klindt et al. [18] who found that creep feeding was only beneficial for larger size litters. It should be noted that, in their study, litters having between 8–14 piglets were considered a large litter. The litter size in the most hyperprolific sow lines has increased from an average of 11.7 live piglets in 2000 to 17.5 in 2019 [51]. Moreover, litters standardized to 14 piglets are not uncommon in commercial pig operations, with attempts to keep two additional piglets in relation to the number of functional teats [52]. However, even in more recent studies (e.g., Boston et al. [15] 2022; Muns et al. [7] 2018; Oliveira et al. [21], 2021), in which modern hyperprolific sows were used, the litter size was standardized to 10–12 piglets. Therefore, more studies evaluating the impacts of creep feeding in larger litters (using ≥14 piglets) are needed.

In the present meta-analysis, no effect of weaning age on creep feed intake was observed. This result is somewhat surprising given the fact that weaning age is considered an important factor affecting creep feed consumption [8]. Additionally, several studies observed that 60 to 80% of the total creep feed consumption takes place in the last week of lactation regardless of weaning age [10,22,24,27,53]. This discrepancy in results could be attributed to the multifactorial aspect of creep feed consumption. Increasing the weaning age may result in increased creep feed consumption within a study; however, when results from different studies are combined, it may not be possible to exclude all factors that can impact creep feed intake (e.g., creep feed complexity, physical form of the creep diet, pellet size, type of creep feeder used, time of onset, creep feed freshness, and sow’s milk production). This emphasizes the importance for studies evaluating the effects of creep feeding to control the variables that can potentially influence creep feed intake.

Creep feeding of suckling piglets is believed to ease the weaning transition by improving feed intake in the immediate post-weaning period [54]. This occurs because the ingestion of creep feed may hasten the induction of digestive enzymes (i.e., amylase and protease) associated with carbohydrate and protein digestion [9]. Piglets that are known creep feed eaters have higher feed intake and better performance after weaning [10]. Consistent with the literature, our results demonstrate that creep feeding in lactation improves piglet post-weaning growth performance, underscoring the importance of creep feed consumption. It should be mentioned that in some studies, the increase in feed intake was not accompanied by increased weight gain. However, as stated by Solà-Oriol and Gasa [8], the parameters related to growth performance may not be the most appropriate ones to better interpret the benefits of creep-feeding. For instance, Muns and Magowan [7] reported that the creep feed offered during lactation had no effect on piglet growth performance after weaning. Nonetheless, piglets that ate creep feed during lactation had a higher average daily feed intake during the first week after weaning than piglets not offered creep feed. Considering that low feed intake or no feed consumption often occurs shortly after weaning, and it can contribute to gut dysfunction [2], an early resumption of feed consumption has an important role to play in mitigating the adverse effects of weaning stress [1]. Of particular interest, there is evidence that creep feeding in lactation improves gut structure after weaning. Kuller et al. [55] reported that piglets that consumed creep feed in lactation had increased small intestine net absorption after weaning. Likewise, the small intestines of creep-fed piglets had higher weights, deeper crypts, and increased cell-proliferation rates [40]. Unfortunately, due to a limited number of studies, we could not evaluate the effects of creep feed consumption on gut structure. Thus, further studies evaluating such effects are warranted.

Our systematic review and meta-analysis confirmed that the practice of creep feeding piglets in lactation positively affects pre- and post-weaning growth performance. However, these results should be seen in light of some potential limitations. Several factors that can influence creep feed consumption could not be evaluated in the present meta-analysis due to variability and (or) lack of information among studies. This precluded that regression analysis could be performed in order to identify the factors that generate a great percentage of creep feed consumers. Likewise, due to the high variability among studies regarding the time in which piglets were weighed in the nursery phase, the effects of creep feeding on piglet post-weaning performance could not be evaluated in the meta-analysis. Furthermore, a great number of studies (48 studies) were not included in the present work due to the absence of a control group without creep feed provision. This contributed to reducing the power of our analysis. Therefore, further trials with high-standardized experimental protocols are necessary to investigate strategies that create a larger proportion of creep feed eaters.

## 5. Conclusions

Creep feeding is a management strategy that positively impacts piglet pre- and post-weaning performance. Collectively, the results of the present work demonstrated that piglets provided with creep feed while nursing had increased body weight at weaning and post-weaning. Based on the present results, our recommendation is that creep feed should be consumed for a minimum of 14 days to improve pre-weaning performance. Unfortunately, in the present work, due to great variability in study protocols and (or) lack of information among studies, the strategies that increase the proportion of creep feed consumers could not be evaluated; this also precluded the evaluation of the creep feeding effects on post-weaning performance in the meta-analysis. Therefore, further trials with high standardization are necessary to investigate methods affecting creep feed consumption and further confirm the beneficial effects of creep feeding.

## Figures and Tables

**Table 1:** Summary of the main outcomes from studies that evaluated the effect of creep feeding on piglet pre- and post-weaning performance.

Variable	Effect	No. of Studies ^3^
Pre-weaning performance ^1^ (Average daily gain and body weight)	Improved	11
	Impaired	0
	No effect	13
post-weaning performance ^2^ (Average daily gain, feed intake, and body weight)	Improved	11
	Impaired	0
	No effect	8
Performance at growing/finishing phase (Average daily gain, feed intake, and body weight)	Improved	0
	Impaired	0
	No effect	5

^1^ The average of the litter was considered to evaluate the effect of creep feeding. The evaluation of eaters or non-eaters was not considered in this evaluation. ^2^ Improved or impaired effect of creep feeding on post-weaning performance was considered if the effect was observed in any period of the post-weaning evaluation. ^3^ The number of studies in the table could be higher than the number of studies included in the systematic review and meta-analysis due to the fact that, in some of the articles, more than one experiment was conducted.

**Table 2:** Summary of the main features and outcomes of studies that evaluated the effects of creep feeding on piglet pre- and post-weaning performance.

Reference	No. of Litters	Diet Form	Energy Content Creep Diet ^1^ (s)	CF Duration	Time of Onset	Weaning Age	Weight at Weaning (kg)	Weight at Nursery ^2^ (kg)	Feed Additives
Bruininx et al. [10]	Creep: 16 No creep: 5 l	Pellet	2370 NE, kcal/kg)	17 d	11 d	28 d	NS	NS	None
Lawlor et al. [34]	Creep: 30 No creep: 30	Pellet	3847 DE, kcal/kg	17 d	11 d	28 d	Creep: 8.5 ^a^ No creep: 7.95 ^b^	NS	None
Sulabo et al. [12]	Creep: 39 No creep: 39	Pellet	3495 ME, kcal/kg	19 d	3 d	21 d	NS	NS	None
Lemieux et al. [35]	Creep: 7–8 No creep: 7–8	NP	NP	16 d	5 d	21 d	NS	NE	4% of dried brewers yeast
Van der Meulen et al. [36]	Creep: 4 No creep: 4	Pellet	2342 NE, kcal/kg	16 d 30 d	12 d	28 d 49 d	*Wean 4 weeks* Creep: 6.0 ^a^ No creep: 5.8 ^a^ *Wean 7 weeks* Creep: 12.8 ^b^ No creep: 11.7 ^c^	NE	None
Bandara et al. [33]	Creep: 55 No creep: 52	NP	NP	7 d	19 d	26 d	NS	NS	None
Yan et al. [20]	Creep: 10 No creep: 10	NP	5000 DE, kcal/kg 4000 DE, kcal/kg	16 d	5 d	21 d	NS	NS	None
Yan et al. [37]	Creep: 8 No creep: 8	NP	4000 DE, kcal/kg	16 d 11 d 6 d	5 d 10 d 15 d	21 d	NP ^2^	NP ^3^	None
Shea et al. [32]	Creep: 40 No creep: 40	NP	NP	7 d	14 d 21 d	21 d 28 d	NS	Creep: 19.90 ^a,^** No creep: 18.64 ^b,^**	None
Cabrera et al. [6]	Creep: 15 No Creep: 45	Pellet	3520 ME, kcal/Kg	7 d	14 d	21.3 d	NS	NS	1% Glutamine 0.88% AG ^4^
Park et al. [38]	Creep: 20 No creep: 20	Liquid	3900 DE, kcal/kg	17 d	4 d	21	Creep: 5.37 ^a^ No creep: 4.82 ^b^	NS	NP
Tran et al. [39]	Creep: 8 No Creep: 8	NP	3471 ME, kcal/kg 3539 ME, kcal/kg	14 d	7 d	23.5 d	NS	Exp creep:13.67 ^ab^ Con creep: 12.48 ^b^ No creep: 13.29 ^b^	10% yeast-dried milk
De Greeff et al. [40]	Creep:5 No creep: 5	Liquid milk replacer ^5^	3444 NE, kcal/kg	19 d	2 d	21 d	NS	NS	None
Balamuralikrishnan et al. [41]	Creep: 80 No creep: 80	NP	3537 ME, kcal/kg	12 d 18 d	9 d	21 d 27 d	*Wean 21* Creep: 6.57 ^a^ No creep: 5.86 ^b^ *Wean 27* Creep 27: 7.84 ^c^ No creep 27: 6.87 ^d^	*Wean 21* Creep: 22.56 ^a^ No creep: 20.28 ^b^ *Wean 27* Creep: 24.05 ^c^ No creep: 21.78 ^d^	None
Lee and Kim [11]	Creep: 4 No creep: 4	NP	4000 DE, kcal/kg	17 d 10 d 3 d	7 d 14 d 21 d	24 d	Creep 7 d: 9.1 kg ^a^ Creep 14 d: 8.5 kg ^b^ Creep 21 d: 8.2 kg ^c^ No creep: 7.8 kg ^c^	NE	None
Muns et al. [7]	Creep: 22 No creep: 29	NP	3943 DE, kcal/kg	10 d	18 d	28 d	NS	NS	None
Oliveira et al. [21]	Creep: 15 No creep: 15	NP	3600 ME, kcal/kg	11 d	10 d	21 d	Front feeder: 5.04 ^a^ Back feeder: 4.63 ^b^ No creep: 4.73 ^b^	NE	None
Middelkoop et al. [42]	Creep: 12 No creep: 10	Pellet	2820 NE, kcal/kg	28 d	2 d	30 d	NS	NS	Non-starch polysaccharides
Martins et al. [43]	Creep: 6 No creep: 5	Gruel Mash	4295 ME, Kcal/kg	18 d	3 d	21 d	NS	NS	None
Sands et al. [44]	Creep: 12 No creep: 14	Mash	2400 NE, kcal/kg 2450 NE, kcal/kg	14 d	14 d	28 d	NS	NS	None

Note: CF, creep feeding; NP, not provided; NS, not significant; NE, not evaluated. ^a,b,c,d^ Different superscript letters denotes statistical significance found in the original studies. ^1^ Standardize to kcal/kg. ^2^ Post-weaning piglet body weight at the end of the experimental period (nursery phase); the time at which piglets were weighed varied among studies. ^3^ Yan et al. [37] provided the average daily gain of treatments during the experimental period without providing the body weight at birth or at weaning. Therefore, it was not possible to calculate the body weight of piglets in each treatment. ^4^ AminoGut is a commercial dietary supplement containing a mixture of L-glutamine (min 10%) and L-glutamate (min 10%). ^5^ Complex milk replacer (Milkiwean Yogurt): whey protein concentrate, whey powder, whey fat kernel, starch, pea protein, wheat gluten isolate, dextrose, yeast, acidifier, AA, and premix (per kg complete diet: 40,000 IU vitamin A, 5000 IU vitamin D3, 300 IU vitamin E, 60 mg Fe, 140 mg Cu, 45 mg Mn, 84 mg Zn, 1 mg I, and 0.3 mg Se). ** Shea et al. [32] used a 2 × 2 factorial design where creep feed provision and weaning age were the factors. However, the authors did not provide average body weight at weaning from the piglets weaning at 21 and 28 days of age. Instead, an average of these two weaning ages was provided and included in the column of weaning weight and final weight.

**Table 3:** Descriptive data of the 20 studies included in the meta-analysis.

Variable	Mean ± SD (Min/Max)
Weaning age (days)	23.6 ± 3.24 (18.9/19.9)
Litter size (*n*)	11.0 ± 1.28 (7.4/13.6)
Piglet body weight at weaning (kg)	6.81 ± 1.23 (4.63/9.80)
Litter weight at weaning (kg)	76.8 ± 16.6 (44.2/111.7)
Average piglet daily gain (g)	267.4 ± 68.2 (158.0/463.0)
Mortality (%)	5.3 ± 4.3 (1.0/15.6)
Age piglets were started on creep feed (days)	10.1± 5.5 (1/21)
Duration of creep feeding (days)	13.4 ± 5.0 (3.0/27.9)
Average litter daily feed intake (g)	269.5 ± 172.1 (24.1/633.35)

**Table 4:** The effects of creep feeding on the litter weight and average piglet body weight at weaning (Mean ± SEM).

Variable	Creep	No-Creep	*p*-Value
Litter weight at weaning (kg)	81.2 ± 4.18	76.4 ± 4.22	<0.001
Average piglet body weight at weaning (kg)	7.23 ± 0.30	6.96 ± 0.31	0.03

**Table 5:** The association of the average piglet body weight and litter weight (dependent variables) at weaning with total creep feeding intake during lactation (Fixed factor).

Item	Average Piglet Body Weight at Weaning			
	Estimate	SEM	*p*-Value	Confidence Interval ^1^
Intercept	6.28	0.38	<0.001	5.48/7.05
Total creep feeding disappearance (kg)	0.22	0.05	<0.001	0.02/0.35
	**Litter Weight at Weaning**			
	**Estimate**	**SEM**	***p*** **-Value**	**Confidence Interval ^1^**
Intercept	70.01	4.73	<0.001	59.97/79.85
Total creep feeding disappearance (kg)	3.13	1.27	<0.001	1.63/4.74

The code of the study and the weaning age were included as random effects in both statistical models. ^1^ Lower and upper limit.

## Data Availability

The digital datasets and analyses of the present study are available from the corresponding author upon reasonable request.

## Supplementary Materials

The following supporting information can be downloaded at: https://www.mdpi.com/article/10.3390/ani13132156/s1, Table S1: Scores for each scientific criteria of the articles selected. Figure S1: Non-linear generalized model used to predict the minimum duration of creep feeding (days) necessary to increase litter weight at weaning.
