# Supplementary material for: A Systematic Review and Meta-Analysis of Creep Feeding Effects on Piglet Pre- and Post-Weaning Performance
Source: Animals (Basel). 2023 Jun 30;13(13):2156. doi: 10.3390/ani13132156 (PMC10340024; doi:10.3390/ani13132156)
Supplement: Supplementary file 1 [file animals-13-02156-s001.zip › animals-2446028-Figure S1.pdf]

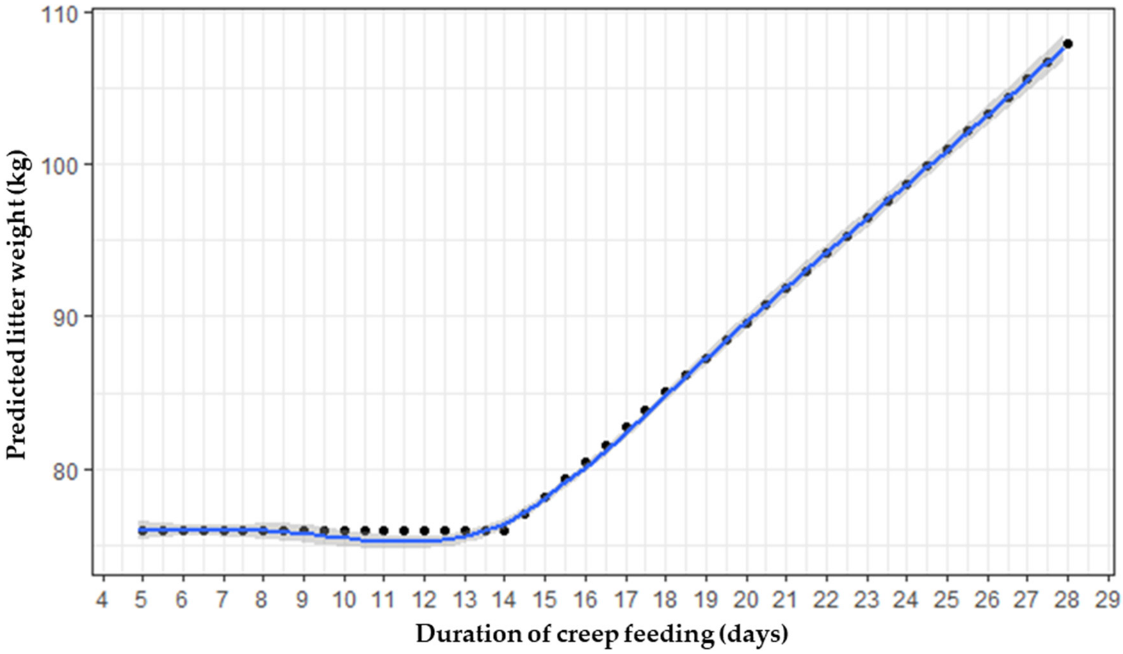

Figure S1. Non-linear generalized model used to predict the minimum duration of creep feeding (days) necessary to increase litter weight at weaning.
