# Supplementary material for: A Systematic Review and Meta-Analysis of Creep Feeding Effects on Piglet Pre- and Post-Weaning Performance
Source: Animals (Basel). 2023 Jun 30;13(13):2156. doi: 10.3390/ani13132156 (PMC10340024; doi:10.3390/ani13132156)
Supplement: Supplementary file 1 [file animals-13-02156-s001.zip › animals-2446028-Table S1.pdf]

Table S1. Scores for each scientific criteria of the articles selected.

Reference	A	B	C	D	E	F	G	H	I	J	K	L	Total
**Sulabo et al. [12]**	2	2	1	2	2	1	2	2	2	2	2	2	23
**Cabrera et al., [6]**	2	1	1	2	2	1	1	2	2	2	2	2	21
**Bruininx et al. [10]**	1	2	1	1	1	1	2	1	1	2	1	2	17
**Park et al. [38]**	2	2	1	2	1	1	1	1	2	2	1	2	19
**Tran et al. [39]**	2	2	1	2	2	1	2	2	2	2	1	2	21
**Martins et al. [43]**	2	2	1	2	1	1	1	2	2	2	2	2	20
**Muns et al. [7]**	1	2	1	2	1	2	2	1	1	2	1	2	19
**Lemieux et al. [35]**	2	2	1	2	2	2	1	1	1	2	1	1	18
**Lawlor et al. [34]**	1	2	1	1	1	1	2	1	1	2	2	1	15
**Lee and Kim [11]**	2	2	1	2	2	1	1	1	2	2	1	1	18
**De Greef et al., [40]**	1	2	1	2	2	1	1	1	2	2	1	1	17
**Balamuralikrishnan et al. [41]**	2	2	1	2	2	1	1	1	2	2	2	1	19
**Yan et al. [20]**	2	2	1	2	2	1	1	1	2	2	2	1	19
**Oliveira et al. [21]**	2	2	2	2	2	2	1	2	2	2	1	1	22
**Yan et al. [37]**	2	2	1	2	2	1	1	1	2	2	2	1	19
**Shea et al. [32]**	1	1	1	2	1	1	2	1	2	2	1	2	17
**Bandara et al. [33]**	1	1	1	2	1	1	1	1	2	2	1	2	16
**Middelkoop et al. [42]**	1	2	1	2	2	2	2	1	2	2	2	2	21
**Van der Meulen et al. [36]**	1	2	1	2	2	1	1	1	1	2	2	2	18
**Sands et al. [44]**	2	2	1	2	1	2	1	1	1	2	2	2	19

A. Randomization: randomized studies scored 2, whilst a non-randomized studies, or when this was not clear stated, scored 1; **B**. Breed or genetic line: studies that mentioned breed or genetic line received a score of 2, and when it was not clearly stated, scored 1; **C**. Environmental characterization: studies that provided information regarding to temperature and humidity in the farrowing room scored 2, and those that did not described it scored 1; **D**. Litter size: studies that described the mean litter size in each experimental group scored 2, and those that did not mention it scored 1; **E**. Composition of the creep diet: studies that detailed the ingredients and the nutritional composition of the creep diet score 2, and those that did not provide this information scored 1; **F**. Creep feeder placement: studies that described where the feeder was

located in the farrowing crate scored 2, and those that did not provide this information scored 1; **G**. Type of creep feeder: studies that described the type of creep feeder scored 2, and those that did not describe it scored 1; **H**. Sample size: studies using more than 30 sows per treatment scored 2 and those using less than 30 sows per treatment scored 1; **I**. Parity: studies that specified parity scored 2, while those that did not clearly mention it scored 1; **J**. Piglet body weight at the start of the experiment: studies that stated the piglet body weight at the start of the experiment scored 2, and those that did not scored 1; **K**. Post weaning performance: studies that evaluated piglet post weaning performance scored 2, and those that did not scored 1; **L**. Physical form of the creep feed: trials that described the physical form of the creep feed scored 2; and trials that did not describe it scored 1;
